# Chronic inhibition of endoplasmic reticulum calcium-release channels and calcium-ATPase lengthens the period of hepatic clock gene *Per1*

**DOI:** 10.1186/1740-3391-9-6

**Published:** 2011-07-08

**Authors:** Adrián Báez-Ruiz, Mauricio Díaz-Muñoz

**Affiliations:** 1 Departament de Neurobiología Moleculary Celular, Instituto de Neurobiología, UNAM-Juriquilla, Boulevard Juriquilla #3001, Apdo. Postal 1-1141, Querétaro, QRO. 76230, México

**Keywords:** Food-entrainable oscillator, liver explants, clock proteins, intracellular calcium, SERCA, IP_3_R, RyR.

## Abstract

**Background:**

The role played by calcium as a regulator of circadian rhythms is not well understood. The effect of the pharmacological inhibition of the ryanodine receptor (RyR), inositol 1,4,5-trisphosphate receptor (IP_3_R), and endoplasmic-reticulum Ca^2+^-ATPase (SERCA), as well as the intracellular Ca^2+^-chelator BAPTA-AM was explored on the 24-h rhythmicity of the liver-clock protein PER1 in an experimental model of circadian synchronization by light and restricted-feeding schedules.

**Methods:**

Liver explants from *Period1-luciferase *(*Per1-luc*) transgenic rats with either free food access or with a restricted meal schedule were treated for several days with drugs to inhibit the activity of IP_3_Rs (2-APB), RyRs (ryanodine), or SERCA (thapsigargin) as well as to suppress intracellular calcium fluctuations (BAPTA-AM). The period of *Per1-luc *expression was measured during and after drug administration.

**Results:**

Liver explants from rats fed ad libitum showed a lengthened period in response to all the drugs tested. The pharmacological treatments of the explants from meal-entrained rats induced the same pattern, with the exception of the ryanodine treatment which, unexpectedly, did not modify the *Per1-luc *period. All effects associated with drug application were reversed after washout, indicating that none of the pharmacological treatments was toxic to the liver cultures.

**Conclusions:**

Our data suggest that Ca^2+ ^mobilized from internal deposits modulates the molecular circadian clock in the liver of rats entrained by light and by restricted meal access.

## Background

In most species, numerous physiological processes are regulated by a timing system associated with the rotational movement (~24 h) of our planet. The circadian rhythms are anticipatory adjustments of metabolic and behavioral processes elicited by daily environmental fluctuations [1]. At the cellular level, circadian rhythms are maintained by a system of interlocking positive (CLOCK and BMAL) and negative (PER and CRY) feedback loops of transcription/translation driven by the core clock genes [2]. The circadian clock, in turn, communicates rhythmicity to a number of output pathways by controlling E-box-associated gene expression and the metabolic status of the cell [3].

Environmental factors that provide entraining cues (zeitgebers) communicate the passage of time to cellular circadian clocks. Two well-recognized zeitgebers are the alternating light/dark cycle and food availability. Photic stimuli entrain the main mammalian pacemaker, the suprachiasmatic nucleus (SCN). It is now well established that restricted feeding also synchronizes rhythmic processes through activation of a food-entrainable oscillator (FEO). The anatomical substrate for the FEO and its dependence on canonical clock gene function remain topics of debate [4,5]. However, two aspects are accepted: 1) the FEO is not completely dependent on the SCN [6]; 2) it requires food restriction (FR) with circadian entraining limits (~22-26 h) [7]. Activation of the FEO influences specific brain areas related to feeding and metabolic control [8] and peripheral tissues such as the liver, which displays a particularly rapid and robust phase shift in response to food entrainment in many physiological and metabolic hepatic functions [9-12]. FR and the associated FEO expression modify not only the phase but also the amplitude of diverse circadian rhythms. For example, notable changes in 24-h variations in the amplitude of free fatty acid concentrations, stomach content, and hepatic triacylglyceride levels have been reported in FR protocols [13-15]. However, it has not been reported if meal entrainment causes modifications in the free-running period of rhythmic parameters.

Compelling evidence for a link between the molecular clock and metabolic networks has been reviewed during light and food entrainment [16-19]. We suggest that intracellular calcium is a strong candidate to coordinate the circadian timing system and biochemical reactions due to its ubiquitous role as a metabolic regulator [20]. Fluctuations of cytoplasmic calcium codify a message that is interpreted in a spatio-temporal manner by metabolic and transcriptional factors. The handling of intracellular calcium is the result of a coordinated activation of ion channels, ATPases, and exchangers in organelles such as the endoplasmic reticulum, mitochondria, and nucleus [21]. Important elements involved in intracellular calcium dynamics include the calcium-release channels inositol 1,4,5-trisphosphate receptor (IP_3_R) and ryanodine receptor (RyR), as well as the sarco-endoplasmic reticulum calcium ATPase (SERCA). Although all of these proteins are expressed and widely studied in the liver [22], the precise regulatory role for calcium dynamics in the function of the molecular clock in the liver has not been described.

The aim of this study was to explore the rhythmic properties of *Per1-Luc *in liver explants (mainly the period) exposed for several days to drugs that specifically alter calcium dynamics in the endoplasmic reticulum. Experiments were conducted using rats fed ad libitum (AL) or under a restricted feeding (FR) schedule. Our data reveal that targeted disruption of calcium dynamics affects circadian rhythms of *Per1-luc *expression in a manner that does not depend on the entrainment cue (meal vs. light), except for the case of ryanodine treatment which, surprisingly, did not alter *Per1-luc *rhythmicity in the FR group.

## Methods

### Animals

Male transgenic rats bearing a *Period1 *gene whose promoter region is linked to the coding sequence of the luciferase gene were used as experimental subjects (*Per1-luc*) [23]. Transgenic *Per1-luc *rats were bred and raised in the vivarium of the Biology Department at the University of Virginia under standard conditions (12:12 h light/dark with lights on as Zeitgeber Time 0 or ZT0) with ad libitum access to food and water. At 5-6 weeks of age (~200 g), animals were transferred to a light- and temperature-controlled cabinet for 5 days prior to food restriction. On day 6, rats were separated into two groups: one group continued with ad libitum feeding (AL), whereas a second group was given a restricted meal (FR from ZT4 to ZT6) for 10 days. All animals were sacrificed on day 15, and liver tissue was recovered for explant culture as described below. All procedures were done according to the National Institutes of Health Guidelines for the Care and Use of Animals, and the Guidelines of the Animal Care and Use Committee at the University of Virginia, USA.

### Locomotor activity recording

Total locomotor activity was monitored with infra-red motion detectors (IR; Quorom RR-150) mounted ~13 cm above each cage. Each IR detector was modified such that a delay of 8-12 s occurred between detected events. Data were collected in 1-min bins (~5-7 detections/min) with ClockLab software (Actimetrics, USA), compressed into 5-min bins, and finally actograms were generated offline with the same software.

### Tissue culture and bioluminiscence recording

Rats were sacrificed on day 15, and hepatic tissues were isolated and cultured as previously described [23]. Briefly, a fraction of one hepatic lobe was quickly removed and chilled in Hanks Balanced Salt Solution (HBSS) at ~4°C. Small explants of liver tissue (1-2 mm^2^) were dissected free from the larger fragment, placed on culture membranes (Millicell-CM; Millipore), and sealed in a glass-covered (Circle #0, Fisher Scientific), 3.5-ml petri dish (BD-Falcon) containing 1.2 ml of culture medium [serum-free, low sodium-bicarbonate, no phenol red, Dulbecco's modified Eagle's medium (Gibco) supplemented with 10 mM HEPES (pH 7.2), 2% B27 (Invitrogen), 0.1% Luciferin (Promega), and antibiotics (25 U/ml penicillin and 25 μg/ml streptomycin; Gibco). Tissue bioluminescence was measured at 1-min intervals with photomultiplier tubes (Hamamatsu Corporation, USA) housed in a light-tight chamber maintained at 36°C. Each explant was kept 1 day without any manipulation before the addition of drugs. Actions and final concentrations of drugs were as follows: BAPTA-AM (membrane-permeable Ca^2+ ^chelator) 30 μM (Sigma, USA); thapsigargin (SERCA antagonist) 1 μM (Calbiochem, USA); aceto-methoxy 2-aminoethoxydiphenyl borate (2-APB; IP_3_R antagonist) 100 μM (Calbiochem, USA); ryanodine (RyR antagonist) 100 μM (Calbiochem, USA), and dimethyl sulfoxide (DMSO) 0.1% as vehicle. Drugs were applied continuously from day 2 to 6 (in a chronic manner). On day 7, drugs were removed, fresh medium was added, and bioluminescence was recorded for an additional 3-4 days (washout). The doses of all these well-characterized drugs were consistent with those employed in *in vivo *and *ex vivo *experimental systems [24-26].

### Data Analysis

Data analysis was performed using the Lumicycle Data Analysis Software (version 2.1, 2008) and OriginPro (version 7.0, 2002). Raw data were detrended and smoothed as described previously [27]. The period (τ) of *Per1-luc *expression was calculated using a χ-square periodogram analysis of 24-h cycles during drug treatments (days 3-6) and after washout (days 8-11) [28]. The periods during treatment and after washout were calculated separately, omitting the transient gap that resulted during the washout handling. These data have been excluded from the Figures to facilitate the analysis of representative traces.

### Statistical Analysis

All results are presented as mean ± SEM. The effects of feeding condition (AL vs. FR) and culture treatment (DMSO vs. drug treatments vs. washout) on the *Per1-luc *period (τ) were examined with a two-way ANOVA followed by a Bonferroni *post-hoc *test (significance at *p <*0.01). Statistical analysis was performed using GraphPad Prism software (version 5.0, 2007).

## Results

### Food Anticipatory Activity during restricted meal

**Figure 1:**
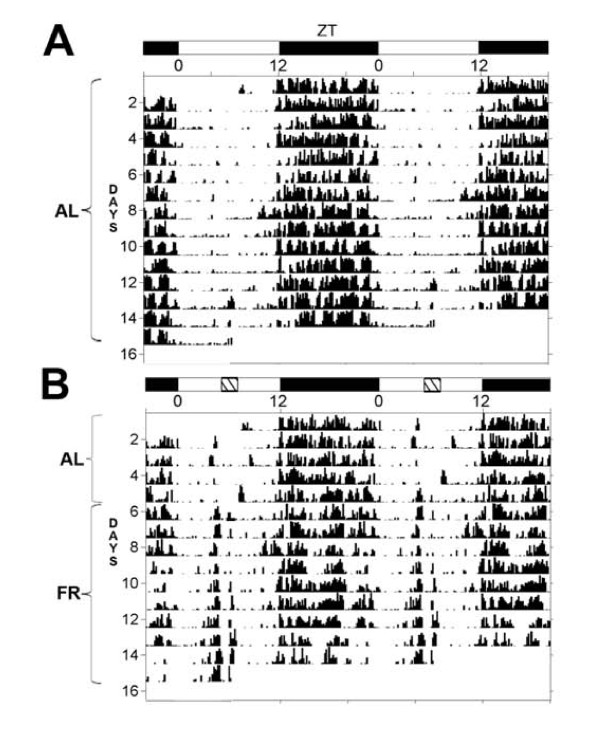
**Representative double-plotted actograms of locomotor activity from *Per1-luc *transgenic rats fed ad-libitum or under restricted feeding in a 12:12 h light:dark cycle**. In the first 5 days all rats were allowed free access to food; one group continued this condition from day 6 to day 15 (A) while another group (B) had a restricted feeding schedule with food available from ZT4 to ZT6 (indicated by the hatched box at the top of the figure inside the bar indicating the L:D cycle). These data are representative of 6 animals.

As expected, representative actograms of locomotor activity revealed the appearance of robust food anticipatory activity (FAA) when rats had access to a 2-h restricted meal for 10 days beginning on day 3 (Figure 1B). These data agree with previous reports and are accepted as evidence of FEO activation in *Per1-luc *transgenic rats [29].

### The meal-entrained phase of Per1-luc expression is maintained in liver explant cultures

**Figure 2:**
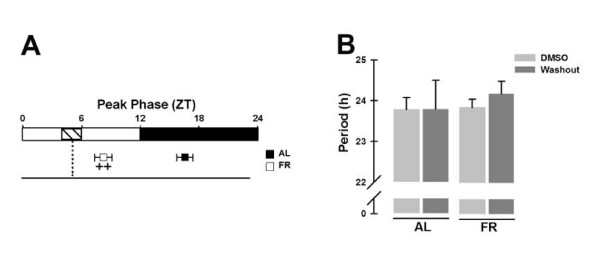
***Per1-luc *bioluminescence rhythms in liver explants treated with DMSO**. (A) Peak phases (in ZT) from *Per1-luc *expression rhythms calculated from the first peak occurring at the beginning of the culture. The dashed rectangle inside the subjective light period indicates the former food-restricted schedule, and the vertical dotted line represents the former meal time of the FR rats. (B) Period from *Per1-luc *expression in liver cultures from AL (left bars) and FR (right bars) rats during and after (washout) DMSO treatment. All data plotted are presented as means ± SEM, n = 6. ^++^Significant difference between AL vs. FR group on the indicated treatment day (Bonferroni post hoc test, α = 0.05).

As expected [9,29], the liver cultures isolated from AL rats showed a *Per1-luc *peak phase during subjective night, whereas the explants from FR animals showed a *Per1-luc *peak phase close to the time of the former food access (peak phase: AL = ZT 15.7 ± 0.8 h vs. FR = ZT 7.7 ± 0.8 h; Bonferroni post-test *p *< 0.001; Figure 2A). The *Per 1-luc *period (τ) in all these conditions showed a constant value close to 24 h, indicating that DMSO is an appropriate reagent vehicle in this experimental protocol (Figure 2B).

### Chelation of intracellular calcium with BAPTA-AM affected liver Per1-luc rhythmicity

**Figure 3:**
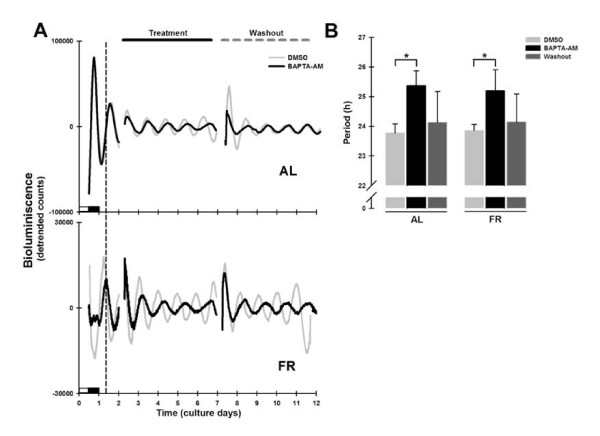
***Per1-luc *bioluminescence rhythms in liver explants treated with BAPTA-AM**. (A) Representative daily records of *Per1-luc *expression in liver explants from AL (superior panel) and FR rats (inferior panel) during BAPTA-AM treatment (100 μM). The grey trace represents the DMSO control and the black trace the BAPTA-AM treated culture. The vertical dotted line represents the former meal time of the FR rats. The horizontal black line above the upper panel indicates the onset and duration of drug treatment which ended with washout of the drug on day 6, marked by the horizontal gray dashed line. The subjective night (or dark phase) is marked only on first day of culture. (B) Period from *Per1-luc *expression in liver cultures from AL (left bars) and FR (right bars) rats during BAPTA-AM treatment and after washout. All data plotted is presented as means ± SEM, n = 6. *Significant difference vs. the DMSO control and ^x ^means difference against washout (Bonferroni post hoc test, α = 0.01).

To determine the role of variations of cytosolic calcium in *Per1-luc *circadian expression, liver cultures were treated with BAPTA-AM. In previous work, the use of this chelator promoted damping of *Per1-luc *rhythm in suprachiasmatic nucleus explants [26]. Representative rhythms of *Per1-luc *expression in liver explants from AL and FR groups treated with BAPTA-AM are shown in Figure 3A. A significant τ lengthening (BAPTA-AM: AL = 25.4 ± 0.5 h and FR = 25.2 ± 0.7 h vs. DMSO: AL = 23.8 ± 0.3 h and FR = 23.9 ± 0.2 h; Bonferroni post-test *p *< 0.001; Figure 3B) was observed when BAPTA treatment was applied to the liver explants in both groups. The BAPTA-AM action was reversed after the change of culture medium (washout: AL = 24.3 ± 1.0 h and FR = 24.1 ± 0.9 h; Figure 3B).

### Thapsigargin altered the period of Per1-luc expression in AL and FR rats

**Figure 4:**
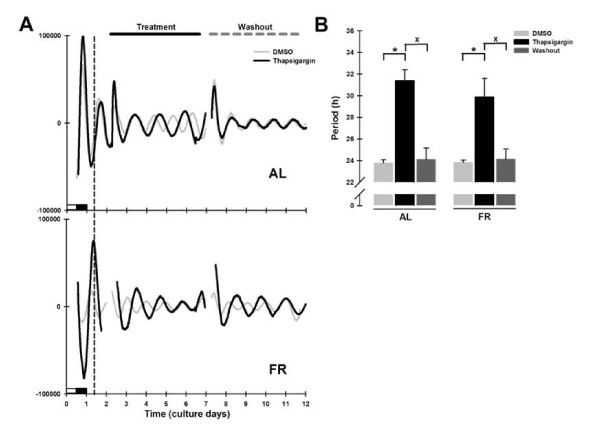
***Per1-luc *bioluminescence rhythms in liver explants treated with thapsigargin**. (A) Representative daily records of *Per1-luc *expression in liver explants from AL (superior panel) and FR (inferior panel) rats during thapsigargin treatment (100 μM). The grey trace represents the DMSO control and the black trace the thapsigargin-treated culture. The vertical dotted line represents the former meal time of the FR rats. The horizontal black line above the upper panel indicates the onset and duration of drug treatment which ended with washout of the drug on day 6, marked by the horizontal gray dashed line. The subjective night (or dark phase) is marked only on the first day of culture. (B) Period from *Per1-luc *expression in liver cultures from AL (left bars) and FR (right bars) rats during thapsigargin treatment and after washout. All data plotted are presented as means ± SEM, n = 6. *Significant difference vs. the DMSO control, and ^x ^means difference against washout (Bonferroni post hoc test, α = 0.01).

Thapsigargin has been widely used as a potent SERCA inhibitor in many types of cells and experimental conditions [30]. Thapsigargin treatment promoted a large increase in the period of *Per1-luc *expression in liver cultures from both AL and FR animals (Figure 4A). Quantification of these actions revealed a significantly lengthened period of *Per1-luc *expression (AL = 31.4 ± 1.0 h and FR = 29.9 ± 1.7 h vs. DMSO; Bonferroni post-test *p *< 0.001; Figure 4B). This effect was eliminated by the washout of the drug in both groups (AL = 24.1 ± 1.0 h and FR = 24.1 ± 0.9 h vs. DMSO; *p *= 0.58; Figure 4B).

### 2-APB altered the period of Per1-luc expression in liver explants from AL and FR rats

**Figure 5:**
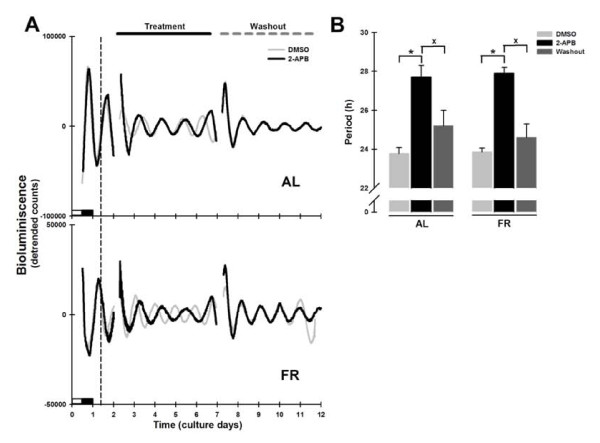
***Per1-luc *bioluminescence rhythms in liver explants treated with 2-APB**. (A) Representative daily records of *Per1-luc *expression in liver explants from AL (superior panel) and FR (inferior panel) rats during 2-APB treatment (100 μM). The grey trace represents the DMSO control and the black trace the 2-APB treated culture. The vertical dotted line represents the former meal time of the FR rats. The horizontal black line above the upper panel indicates the onset and duration of drug treatment which ended with washout of the drug on day 6, marked by the horizontal grey dashed line. The subjective night (or dark phase) is marked only on the first day of culture. (B) Period from *Per1-luc *expression in liver cultures from AL (left bars) and FR (right bars) rats during 2-APB treatment and after washout. All data plotted are presented as means ± SEM, n = 6. *Significant difference vs. the DMSO control, and ^x ^means difference against washout (Bonferroni post hoc test, α = 0.01).

2-APB is commonly used as an inhibitor of IP_3_Rs [25,31]. As was the case with BAPTA-AM and thapsigargin, exposure of liver explants from both AL and FR rats to 2-APB significantly lengthened the period of *Per1-luc *expression (Figure 5A; AL = 27.7 ± 0.6 h and FR = 27.9 ± 0.3 h vs. DMSO; Bonferroni post-test *p *< 0.001; Figure 5B). As with BAPTA-AM and thapsigargin treatments, this effect was abolished when the drug was removed (AL = 25.2 ± 0.8 h and FR = 24.6 ± 0.7 h; *p *= 0.65; Figure 5B).

### Ryanodine lengthened the period of Per1-luc expression in liver explants from AL but not FR rats

**Figure 6:**
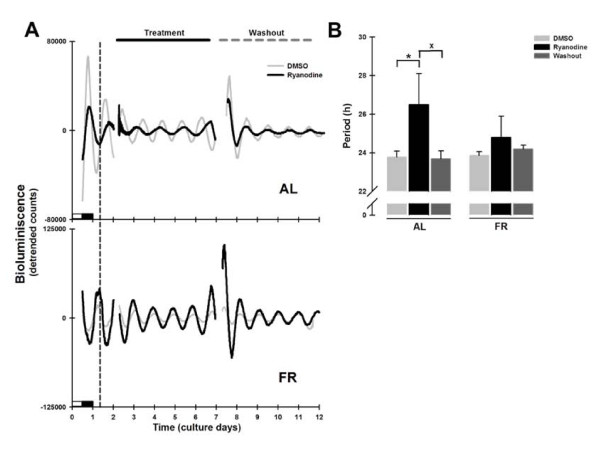
***Per1-luc *bioluminescence rhythms in liver explants treated with ryanodine**. (A) Representative daily records of *Per1-luc *expression in liver explants from AL (superior panel) and FR (inferior panel) rats during ryanodine treatment (100 μM). The grey trace represents the DMSO control and the black trace the ryanodine treated culture. The vertical dotted line represents the former meal time of the FR rats. The horizontal black line above the upper panel indicates the onset and duration of drug treatment which ended with washout of the drug on day 6, marked by the horizontal grey dashed line. The subjective night (or dark phase) is marked only on the first day of culture. (B) Period of *Per1-luc *expression in liver cultures from AL (left bars) and FR (right bars) rats during ryanodine treatment and after washout. All data plotted are presented as means ± SEM, n = 6. *Significant difference vs. the DMSO control, and ^x ^means difference against washout (Bonferroni post hoc test, α = 0.01).

The open probability of the RyR increases in response to ryanodine in the nM range, a state of subconductance is adopted when ryanodine is between 1 and 10 μM, and the channel is inhibited at higher concentration (80-100 μM) [32]. Surprisingly, treatment of the liver cultures with an inhibitory concentration of ryanodine only affected the *Per1-luc *period of the AL group (ryanodine AL = 26.5 ± 1.6 h, AL = 23.8 ± 0.3 h vs. DMSO; Bonferroni post-test *p *< 0.001; Figure 6). The period was longer than that observed for DMSO during treatment, but returned to basal values after washout (washout AL = 23.7 ± 0.4 h, AL = 23.8 ± 0.3 h vs. DMSO; *p *= 0.32; Figure 6B).

## Discussion

We tested the hypothesis that intracellular calcium plays a role in the regulation of the rhythmicity of hepatic *Per1-luc *under *ex vivo *conditions. Our data clearly show that the IP_3_R, RyR, and SERCA inhibitors and intracellular calcium fluctuations altered the daily expression of *Per1-luc *in liver explants. Furthermore, the pharmacological action of ryanodine was dependent upon feeding condition.

It has been reported that intracellular calcium plays diverse roles as a component of the timing system by regulating the entrainment process [25,33], clock gene expression [34,35], and output signaling [36]. Diurnal fluctuations of cytoplasmic calcium [37] as well of RyR and IP_3_R have been observed in the SCN [38,39]. Supporting the importance of intracellular calcium dynamics in the rhythmicity of the SCN, calcium buffering (with concentrations over 40 μM of BAPTA-AM) or low calcium levels (Ca^2+^-free media) promoted damping of *Per1-luc *expression in a dose-dependent manner in SCN explants [26]. In NIH3T3 and rat1-fibroblast cell cultures, thapsigargin and calimycin (a calcium ionophore) increased *Per1 *expression during the first hours of treatment [35,40].

Fluctuations of cytoplasmic calcium codify a message that is interpreted in a spatio-temporal manner by metabolic and transcription factors, thus regulating a variety of cellular processes associated with cell proliferation, apoptosis, biochemical control, and gene expression. This fine-tuned handling of intracellular calcium is the result of a coordinated activation of ion channels, metabolic pumps, and exchangers in organelles such as the endoplasmic reticulum, mitochondria, and nucleus [21]. A role for calcium in the regulation of circadian rhythms and activation of clock-gene proteins has already been reported in protozoa (*Euglena gracilis *and *Paramecium multimicronucleatum*), mollusks (*Bulla gouldiana*) [41-43], plants (*Arabidopsis thaliana*) [44], insects (*Drosophila melanogaster) *[45], and in the mammalian SCN [24,37]. However, the precise regulatory role for calcium dynamics in the function of the molecular clock in the liver has not been described.

The liver is a complex organ formed by several cellular types. Even the most prevalent population, that of hepatocytes, varies with anatomical localization and metabolic performance (periportal and pericentral cells). So far, it is not known if the molecular clock in the liver is equally distributed throughout the entire organ, though our preliminary data in experiments done *in vivo *suggest zone-specific expression of PER1 protein in the hepatic parenchyma (data not shown). The subcellular distribution of the endoplasmic reticulum also changes within the hepatocytes, and potentially the intracellular location of the IP_3_R, RyR, and SERCA. Different isoforms of calcium-release channels are situated in regions of the endoplasmic reticulum next to the plasma membrane (IP_3_R type II), or around the nucleus (IP_3_R type I and RyR) [22]. The heterogeneity of these calcium-handling proteins might underlie a complex interaction between the molecular clock, the intracellular calcium dynamics, and eventually the circadian control of liver metabolic activity. Also, the calcium content of the endoplasmic reticulum is important for the regulation of metabolic pathways, the cell cycle, and cancer progression [46].

The treatments of liver explants from AL and FR rats with BAPTA, 2-APB, and thapsigargin resulted in a lengthening in the *Per1-luc *period. However, the alteration in the period varied from ~1.5 h (with BAPTA-AM) to ~4 h (with 2-APB) and ~7 h (with thapsigargin). Our data strongly suggest that in the liver there exist different types of interactions between the intracellular calcium dynamics and the molecular clock, ranging from mild (related to cytoplasmic calcium levels, with BAPTA-AM), to medium (related to the calcium pool released by IP_3_R, as with 2-apb), and strong (related to the calcium content within the endoplasmic reticulum, as observed with thapsigargin treatment). Therefore, it is well known that these interactions between the handling of intracellular calcium and the molecular clock of the liver are independent of the type of circadian synchronization (by light or by meal) and the feeding protocol (AL or FR). In contrast, the period of *Per1-luc *expression was lengthened during RyR inhibition only in explants from AL animals. No effect was observed when ryanodine was added to explants from FR animals. This fact implies that during the restricted feeding schedule, the period of *Per1-luc *expression is no longer dependent on the calcium released by the RyR. In a different set of experiments, we observed that the 24-h rhythmicity of [^3^H]-ryanodine binding to liver microsomal membranes in the daytime FR protocol showed an increase in amplitude, without change in phase (data not shown). This result suggests that the rhythmic properties of the RyR change as a consequence of FR schedule/FEO expression. In addition, the damping rate and the relative amplitude of the Per1-luc rhythm were not changed by the treatments (data not shown).

The changes in *Per1-luc *period rhythmicity associated with drug treatments could not be interpreted in terms of "after-effects". An "after-effect" is a transient change in the free-running period of an oscillator after a defined period of prior entrainment [47].

The influence of intracellular calcium on liver *Per1-luc *rhythmicity could be explained by transcriptional regulation of the mRNA for this clock gene or by modulation of the phosphorylation status of PER1 protein. For example, a hypo-phosphorylated state of PER1 delays its degradation and slows the rest of the circadian network [48]. As shown previously by Oh-hashi et al. [35], a significant increase in *Per1 *and *Per2 *transcription (also *Cry1 *as discussed in this report) occurred 1 h after NIH3T3 cells were treated with 0.5 μM thapsigargin. A reduction in cytoplasmic calcium availability is expected with all pharmacological treatments, since BAPTA-AM buffers transient elevations of free intracellular calcium concentration, thapsigargin promotes a depletion of the internal calcium deposits, and the inhibition of IP_3_R and RyR hinders the release of calcium. The decrease in intracellular calcium could change *Per1-luc *rhythmicity by modifying the function of other clock genes/proteins such as *Cry1 *or the casein kinase I epsilon (CKIε), which acts as a negative transcriptional regulator of *Per1 *expression or as a PER1-phosphorylating element that promotes proteosomal degradation [48]. Elevation of intracellular calcium concentration and the AMP/ATP ratio activate the 5'-AMP kinase (AMPK), which has been proposed as a metabolic sensor capable of coordinating the circadian clockwork of peripheral oscillators through phosphorylation of *Cry1 *and CKIε, allowing a rapid degradation of *Per1 *expression and shortening of the period [33,49]. It has been suggested that CamKII (calcium/calmodulin-dependent protein kinase II) could mediate the coupling between intracellular calcium levels and the circadian clock, since it promotes the expression of *Per1 *in the SCN in a calcium-dependent process [27,50]. For example, a role for this calcium-sensitive protein in regulating the circadian oscillations driven by CLOCK/CYCLE has been reported in reconstituted tissue cultures from *D. melanogaster *[51]. In addition, there is evidence that buffering intracellular calcium can lengthen the *in vivo *locomotor activity of the fruit fly [50]. These data correlate with our results showing that a decrease in intracellular calcium has a slowing effect on the period.

## Conclusion

Our data support the notion that intracellular calcium dynamics can act as a link between the internal metabolic cues and the circadian clock in the liver. Several drugs that interfere with endoplasmic reticulum-calcium homeostasis slowed the period of the hepatic clock gene *Per1*. We conclude that intracellular calcium dynamics are a key element in the mechanisms that coordinate proper function of the hepatic clock, acting through modification of the timing system in the liver, and in some cases, also according to the feeding condition.

## Competing interests

The authors declare that they have no competing interests.

## Authors' contributions

ABR and MDM designed and conducted the study, analyzed the data, and wrote the manuscript. All authors read and approved the final manuscript.
